# CT radiomics features of meso-esophageal fat in predicting overall survival of patients with locally advanced esophageal squamous cell carcinoma treated by definitive chemoradiotherapy

**DOI:** 10.1186/s12885-023-10973-5

**Published:** 2023-05-25

**Authors:** Shuo Yan, Fei-Ping Li, Lian Jian, Hai-Tao Zhu, Bo Zhao, Xiao-Ting Li, Yan-Jie Shi, Ying-Shi Sun

**Affiliations:** 1 Department of Radiology, Key Laboratory of Carcinogenesis and Translational Research (Ministry of Education/ Beijing), Peking University Cancer Hospital & Institute, No.52 Fu Cheng Road, Hai Dian District, Beijing, 100142 China; 2 Department of Radiology, Hunan Cancer Hospital, The Affiliated Cancer Hospital of Xiangya School of Medicine, Central South University, Changsha, China

**Keywords:** Esophageal squamous cell carcinoma, Tomography, X-ray computed, Chemoradiotherapy, Survival analysis, Radiomics

## Abstract

**Objective:**

To investigate the value of CT radiomics features of meso-esophageal fat in the overall survival (OS) prediction of patients with locally advanced esophageal squamous cell carcinoma (ESCC).

**Methods:**

A total of 166 patients with locally advanced ESCC in two medical centers were retrospectively analyzed. The volume of interest (VOI) of meso-esophageal fat and tumor were manually delineated on enhanced chest CT using ITK-SNAP. Radiomics features were extracted from the VOIs by Pyradiomics and then selected using the t-test, the Cox regression analysis, and the least absolute shrinkage and selection operator. The radiomics scores of meso-esophageal fat and tumors for OS were constructed by a linear combination of the selected radiomic features. The performance of both models was evaluated and compared by the C-index. Time-dependent receiver operating characteristic (ROC) analysis was employed to analyze the prognostic value of the meso-esophageal fat-based model. A combined model for risk evaluation was constructed based on multivariate analysis.

**Results:**

The CT radiomic model of meso-esophageal fat showed valuable performance for survival analysis, with C-indexes of 0.688, 0.708, and 0.660 in the training, internal, and external validation cohorts, respectively. The 1-year, 2-year, and 3-year ROC curves showed AUCs of 0.640–0.793 in the cohorts. The model performed equivalently compared to the tumor-based radiomic model and performed better compared to the CT features-based model. Multivariate analysis showed that meso-rad-score was the only factor associated with OS.

**Conclusions:**

A baseline CT radiomic model based on the meso-esophagus provide valuable prognostic information for ESCC patients treated with dCRT.

**Supplementary Information:**

The online version contains supplementary material available at 10.1186/s12885-023-10973-5.

## Background

Esophageal cancer is the seventh leading cause of tumor-related deaths worldwide [1]. The standard treatment for locally advanced esophageal cancer encompasses multimodal strategies. Neoadjuvant treatment followed by surgical resection is a commonly recommended therapy for T2 or greater disease, or disease with localized metastatic lymph nodes. However, there are groups of patients who cannot benefit from neoadjuvant treatment or tolerate surgery due to poor systemic conditions. It was reported that 6.9%~11.7% of patients could not survive curative resection after neoadjuvant treatment and suffered poor survival [2–4]. In this regard, definitive chemoradiotherapy (dCRT) is an alternative in those cases. However, a study showed that approximately 50% of patients did not respond well to dCRT [5]. Immunotherapy and targeted therapy are new treatments that could be further complementary strategy for improving overall survival in those patients [6–8], however, are of high cost. Early prediction of dCRT outcome offers an opportunity to design an appropriate treatment plan. Radiomics is a tool that extracts high-throughput quantitative features from medical images and provides novel information for tumor evaluation and therapeutic response assessment. It has been extensively used in esophageal cancer for response evaluation [9, 10], prediction of lymph node metastasis [11], tumor stage [12], and survival analysis [13–17]. Most of the previous radiomics studies in esophageal cancer have mainly focused on the intra-tumoral region and/or the lymph nodes [10–18].

Recently, molecular evidence has suggested that the interaction between tumor cells and peritumoral adipocytes plays a critical role in tumor growth and metastases. Peritumoral adipose tissue cells can facilitate cancer initiation, progression, metastasis, and therapeutic resistance through adipokines and also provide energy sources [19, 20]. Esophageal cancer tumor cells tend to spread longitudinally and interact with the lymphatic plexus along the whole length of the esophagus [21, 22], which makes it difficult for medical imaging to determine the extent of local spread of cancer. Meso-esophagus is a newly described structure that surrounds the entire length of the thoracic esophagus and contains fat and esophageal lymph vessels and nodes, faced by a thin layer of connective tissue [23]. The total meso-esophagus excision improves local control of esophageal cancer [24] and is a standard surgical procedure. We speculate that the meso-esophagus might contain information about tumor spread and that the fat in the meso-esophagus may interact with tumor cells along the esophagus in this type of cancer.

Thus, we aimed to develop and validate a CT-based model using radiomic features of meso-esophageal fat to predict overall survival (OS) in locally advanced esophageal cancer treated with dCRT.

## Materials and methods

**Fig. 1:**
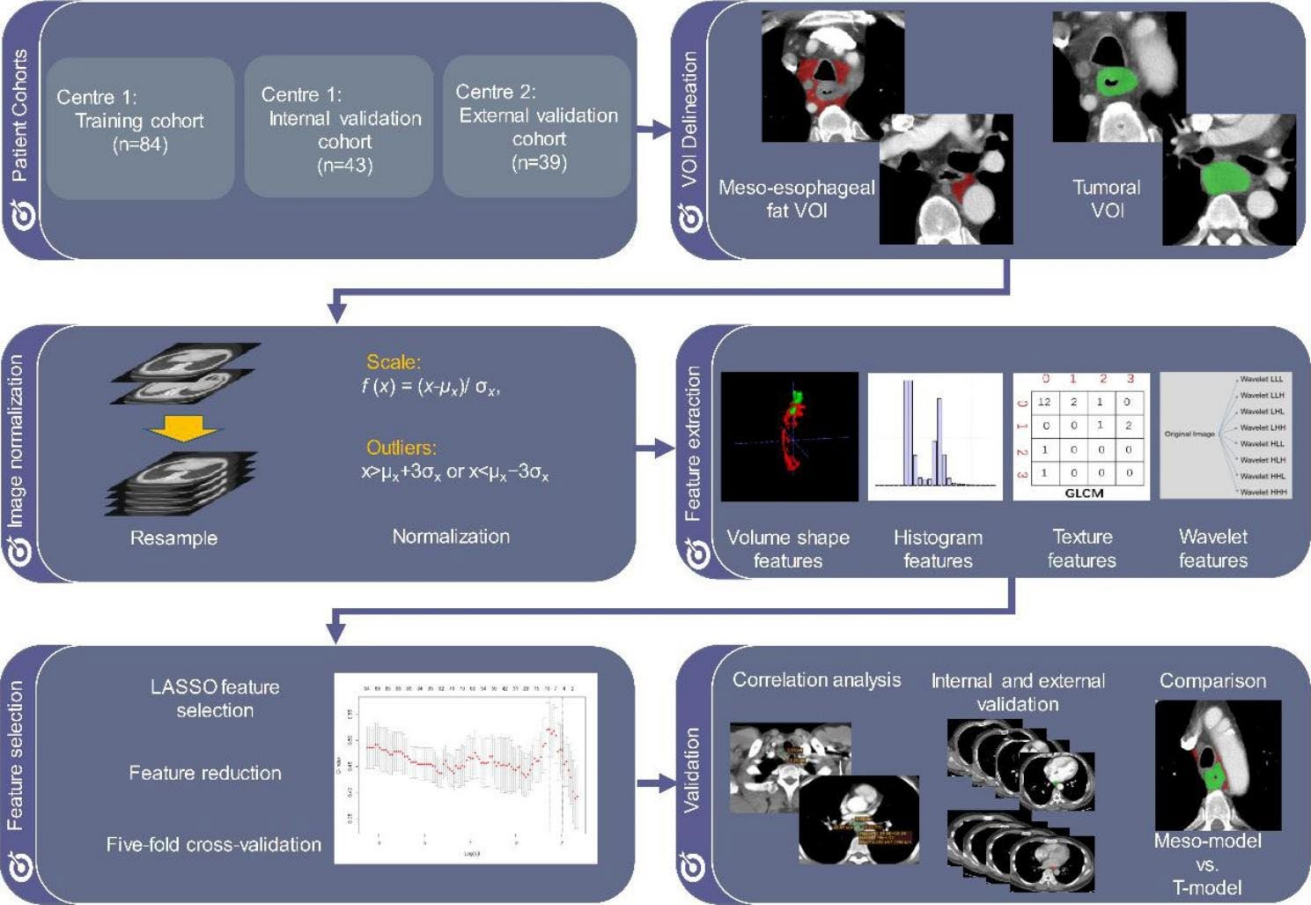
Work Flow of The Study

This study was approved by the Institutional Review Board of Beijing Cancer Hospital (2017KT11) was conducted according to the tenets of the Declaration of Helsinki. Written informed consent was revoked for this retrospective study by IRB of Beijing Cancer Hospital. The study design is depicted in Fig. 1.

### Patient cohorts

Two independent cohorts of esophageal cancer patients from Center 1 and Center 2 were retrospectively identified. Patients who met the following criteria were included: (I) were first diagnosed with primary locally advanced ESCC in the thoracic esophagus, without evidence of distant metastasis; (II) received treatment in the centers; (III) were declared inoperable by a multidisciplinary medical team or refused to undergo surgery; (IV) received dCRT at a dose of 50.4 Gy or more and chemotherapy for 2 cycles or more; (V) had no evidence of organ failure; and (VI) had enhanced chest CT before dCRT. Patients were excluded for the following reasons: (I) failure to complete the full course of dCRT; (II) received esophagectomy after dCRT; (III) history of other malignant tumors; (IV) lost to follow-up in 12 months or death from nontumoral causes; and (V) the image quality of enhanced chest CT was too poor for analysis. A total of 166 patients (127 from Centre 1 and 39 from Centre 2) met the above criteria were included in the study.

Radiotherapy at a dose of 50.4 Gy or more was given in 27–30 fractions for all patients. The majority of the patients in both cohorts received chemotherapy based on a regimen of paclitaxel combined with cisplatin concurrent with radiotherapy and/or as induction therapy; for patients of advanced age or poor physical condition, the treatment involved a reduced dose of chemotherapy or a regimen of paclitaxel.

Patients from Center 1 were randomly allocated to the training and internal validation cohorts in a 2:1 ratio. Patients from Center 2 were used as the external validation cohort.

### CT protocols

The enhanced CT images of Center 1 were obtained via a 64-row helical CT scanner (Lightspeed VCT; General Electrical Medical Systems). The CT images in Center 2 were scanned using a 64-row helical CT scanner (Somatom Definition AS+, Siemens) or a 256-row scanner (256 Revolution; GE Healthcare). The detailed scanning protocols are listed in Supplement Table 1.

### CT feature evaluation

The venous phase of contrast enhanced CT images from both centers were evaluated by a radiologist with 9 years of experience in chest imaging (Reader1, S.Y.) who was blinded to the survival outcomes. The clinical stage of ESCC was assessed according to the guidelines of 8th edition of the Union for International Cancer Control–American Joint Committee on Cancer (UICC-AJCC) tumor, node, metastasis (TNM) staging system based on contrast-enhanced CT images. The criteria for clinical staging of ESCC and quantitative measurement of radiological features are listed in the Supplement Material 1 and 2. The associations between radiological features and OS were assessed by univariate Cox regression analysis. The radiological features with a p<0.10 in univariate regression analysis were included in multivariate Cox regression. Then, a CT features-based model (CT-model) was calculated based on independent risk factors for OS selected by the multivariate Cox regression.

### Segmentation of meso-esophageal fat and tumor

**Fig. 2:**
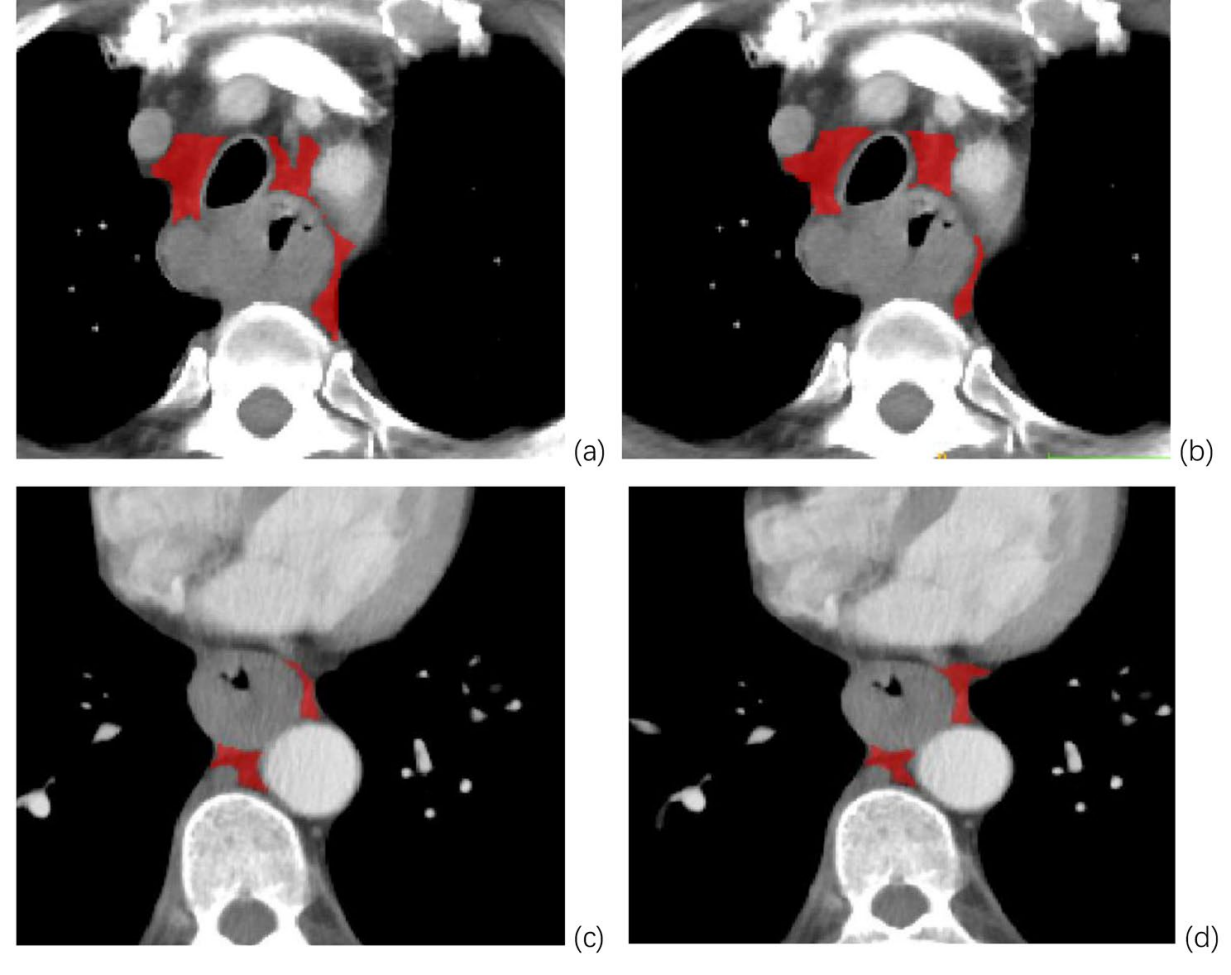
Segmentation of meso-esophageal fat by two radiologists. **a** (by reader 1) and **b** (by reader 2) Segmentation of meso-esophageal fat above the carina. **c** (by reader 1) and **d** (by reader 2) Segmentation of meso-esophageal fat for the infra carinal esophagus

The VOIs of meso-esophageal fat and primary tumor were manually delineated on enhanced CT images using the ITK-SNAP image segmentation software version 3.8 (http://www.itksnap.org). Enhanced images of venous phase were used based on the fact that tumor, lymph nodes and vessels could be demonstrated clearly in the phase [25], which facilitated the boundary of the delineation. Twenty-eight cases from Center 1 were randomly selected and delineated by two radiologists with 9 years (Reader1, S.Y.) and 5 years of experience (Reader2, B.Z.) in thoracic imaging to detect features with good inter-reader agreement. Then all the cases were segmented by a radiologist with 9 years of experience (Reader1, S.Y.). Tumoral VOIs covered the whole volume of the tumor slice by slice, while necrotic areas adjacent to lumen were excluded [26]. Meso-esophagus segmentation was delineated from the level of the sternal notch to the esophageal hiatus, while the esophagus or tumors, lymph nodes, and vessels were excluded. For the esophagus above the carina, the meso-esophagus expanded laterally to the mediastinal structures and forward to the level of the anterior wall of the bronchus [27] (Fig. 2a, b). The anterior wall of the trachea was chosen as the anterior boundary because the anatomical study confirmed that the trachea and esophagus above the level of the carina were co-encased by the mesenteric fascia [27]. For the infra carinal esophagus, the meso-esophagus was found between the left aspect of the esophagus and the descending aorta [23] (Fig. 2c, d). The VOIs of meso-esophageal fat was segmented slice by slice; if there was no periesophageal fat in the abovementioned area, no region was delineated on the slice.

### Feature selection and model construction

CT images were resampled to a resolution of 0.7 mm× 0.7 mm× 0.7 mm through the B-spline interpolation. Normalization was performed by centering the gray level of all voxels at their mean value with standard deviation. A total of 851 features were extracted from the meso-esophageal VOI using the PyRadiomics software (Supplement Material 3 and Supplement Table 2). Features from the 28 subjects delineated by the two radiologists were used to calculate a cross-correlation. Only the features whose cross-correlations were larger than 0.8 were preserved for modeling.

The Cox model was constructed using a linear combination of the features with the least absolute shrinkage and selection operator (LASSO). Briefly, a parameter α was used to control the degree of L1 regularization for feature reduction to avoid overfitting. First, fivefold cross-validation was used in the training group to determine the optimal α value by maximizing the average accuracy. Next, all subjects in the training group were included to train the model again with a fixed α. Finally, the model was evaluated in both an independent internal validation group and an external validation group. For each subject, a radiomics score was provided by the model to evaluate the risk, and was named as meso-rad-score. The model constructed based on meso-esophageal fat radiomics features was named by meso-model. The diagnostic aptitude of the meso-model was evaluated using Harrell’s Concordance Index (C-index) and time-dependent receiver operating characteristic (ROC) curve analysis. A calibration curve was drawn by bootstrapping 1000 samples to evaluate the calibration of the model, along with a Hosmer-Lemeshow fit test.

A tumoral model (t-model) was constructed with tumoral features according to the same work flow as creating the meso-model. We also tried to construct a combined model by combining the CT-model, t-model and meso-model by the multivariable Cox analysis and evaluated the performance of the combined model in the cohorts. The predictive performance of the t-model and CT-model was compared to that of the meso-model and the combined model using the C-indexes in the training, internal validation, and external validation cohorts.

### Follow-up

The patients were regularly followed up with interviews at 2 ~ 6-month intervals during the first year; then, at 6 ~ 12 months intervals the next year and finally every 12 months or more until death. They were also followed up when suffering from worsening symptoms. Systemic physical examinations were performed during follow-ups, and CT examinations were conducted when needed. The dates of first therapy, last follow-up, esophageal cancer-specific death, and disease progression were recorded. OS was measured from the first therapy date until esophageal cancer-specific death. In surviving patients, OS was censored at the last follow-up visit.

### Statistical analysis

Continuous variables were expressed as mean ± standard deviation or median (quartiles), while categorical variables were described as numbers and percentages. Comparisons were conducted using the χ2 or Fisher’s exact test for categorical variables and the T test or Mann-Whitney test for continuous variables. Statistical analysis was performed using SPSS, version 22.0 (IBM), and R statistical software, version 4.2.0 (https://cloud.r-project.org/bin/windows/base/). A 2-tailed p < 0.05 was regarded as significant.

## Results

### Clinical characteristics of patients

The baseline characteristics of patients in the training, internal validation, and external validation cohorts are summarized in Table 1. No significant difference was found in the demographic distributions the groups. The estimated median survival times of the training, internal validation, and external validation cohorts were 39.2 (95% CI: 26.1–52.3) months, 38.1 (95% CI: 29.1–47.1) months, and 22.0 (95% CI: 14.0–30.0) months, respectively. When the three groups were compared, survival analysis revealed no significant difference in survival among them (P = 0.266). During follow-up, 46 (54.8%), 19 (44.2%), and 24 (61.5%) patients died from tumor-specific reasons in the training, internal validation, and external validation cohorts, respectively.

**Table 1:** Patients’ Demographic Information and Radiological Features

Characteristics	Training cohort (n = 84)	Internal validation cohort (n = 43)	External validation cohort (n = 39)	*p*
Age, years (mean ± std)	61.6 ± 7.1	61.6 ± 9.1	60.2 ± 7.6	0.626
Sex, case (%)				0.158
Male	74 (88.1)	33 (76.7)	35 (89.7)	
Female	10 (11.9)	10 (23.3)	4 (10.3)	
BMI (kg/m^2^) (mean ± std)	22.9 ± 3.0	22.8 ± 3.3	22.4 ± 2.7	0.694
Clinical T stage, case (%)				0.229
T2	7 (8.3)	5 (11.6)	8 (20.5)	
T3	50 (59.5)	29 (67.5)	19 (48.7)	
T4	27 (32.2)	9 (20.9)	12 (30.8)	
Clinical N stage, case (%)				0.700
N0	10 (11.9)	9 (20.9)	7 (17.9)	
N1	31 (36.9)	15 (34.9)	11 (28.2)	
N2	38 (45.2)	15 (34.9))	17 (43.6)	
N3	5 (6.0)	4 (9.3)	4 (10.3)	
Clinical stage, case (%)				0.188
II	7 (8.3)	8 (18.6)	9 (23.1)	
III	47 (56.0)	24 (55.8)	18 (46.2)	
IV	30 (35.7)	11 (25.6)	12 (30.7)	
Tumor thickness (mm)	16.6 ± 6.4	15.2 ± 6.7	17.8 ± 6.5	0.188
Tumor length (mm)	48.4 ± 21.1	49.6 ± 18.6	56.5 ± 18.0	0.101
No. of metastatic LN (n, (quartiles))	3 (1,4)	2 (1,4)	2 (1,4)	0.873
LN-long (mm)	22.4 ± 12.0	20.4 ± 11.0	17.0 ± 9.5	0.083
LN-short (mm)	17.1 ± 9.7	15.4 ± 9.4	12.4 ± 7.0	0.058

Note: LN is for lymph node, LN−long is for the long diameter of the lymph node, LN−short is for the short diameter of the lymph node

### CT feature evaluation and modeling

The basic CT features of the ESCC patients in the cohorts are summarized in Table 1. When performing univariate Cox analysis, the tumor thickness and length measured on CT images were considered as predictor for OS (*p* = 0.025 and 0.064<0.10, respectively). The multivariable Cox analysis indicated that tumor thickness was the only factor associated with OS (Hazard ratio, 1.033; 95% CI, 1.004–1.063; *p* = 0.025) (Supplement Table 3). The CT-model was constructed by the single CT feature. It yielded C-indexes of 0.523 (95% CI, 0.402–0.644), 0.502 (95% CI, 0.392–0.612), and 0.498 (95% CI, 0.429–0.567) in the training, internal and external validation cohorts, respectively (Table 2).

**Table 2:** The Performance of CT Features-based Model, Meso-Esophageal Fat-Based, and Tumor-Based Models for OS Evaluation in Patients with ESCC

Models	C-index (95% CI)		
	Training cohort	Testing cohort	External validation cohort
CT features	0.523 (0.402–0.644)	0.502 (0.392-0612)	0.498 (0.429–0.567)
Meso-esophageal fat	0.688 (0.610–0.765)	0.708 (0.598–0.816)	0.660 (0.540–0.781)
Tumor	0.665 (0.589–0.742)	0.602 (0.465–0.740)	0.524 (0.408–0.640)
*p1*	0.312	0.077	0.048
*p2*	0.039	0.002	0.005

Note: *p*1 was calculated from the comparison between meso-esophageal fat-based models and tumor-based models; *p*2 was calculated from the comparison between meso-esophageal fat-based models and CT features-based models

### Meso-esophageal feature selection and radiomic survival prediction model

To predict the OS of patients treated with dCRT, 5 radiomic features in the training cohort were selected through the LASSO method. The meso-esophageal fat radiomic score for each patient was calculated using the linear combination of the features and their corresponding coefficients. For this purpose, the formula below was used:

Meso-rad-score = −5.8860738346 × original_shape_Sphericity − 0.0071604559 ×wavelet-HHH_firstorder_Kurtosis − 0.0003810281 × wavelet-HLL_gldm_LargeDependenceHighGrayLevelEmphasis + 0.0016413261 × wavelet-LLH_firstorder_Kurtosis + 1.2001111039 × wavelet-LLH_gldm_DependenceEntropy.

The meso-model construction was founded on the radiomics score. It indicated favorable performance for OS prediction, with C-indexes of 0.688, 0.708, and 0.660 in the training, internal and external validation cohorts, respectively (Table 2). Higher radiomic scores involved higher risks of death. The results of the time-dependent ROCs are shown in Table 3.

**Table 3:** Areas Under The ROC Curves for 1-Year, 2-Year, and 3-Year Survival Prediction Using Meso-Esophageal Fat Radiomics Model

	1-year	2-year	3-year
Training cohort	0.677	0.731	0.692
Internal validation cohort	0.640	0.793	0.740
External validation cohort	0.721	0.672	0.646

### Comparison with the t-model and CT features

A total of 4 radiomic features proved statistically significant in the univariate analysis. The detailed radiomic score (t-rad-score) for the t-model is presented in the Supplement Material 3. The final predictive model with 4 radiomic features showed slightly lower C-indexes than that of the meso-model in the training and internal validation cohort, without significant difference; and significantly lower C-index in the external validation cohort (Table 2) (*p* = 0.048).

### Combined model

**Fig. 3:**
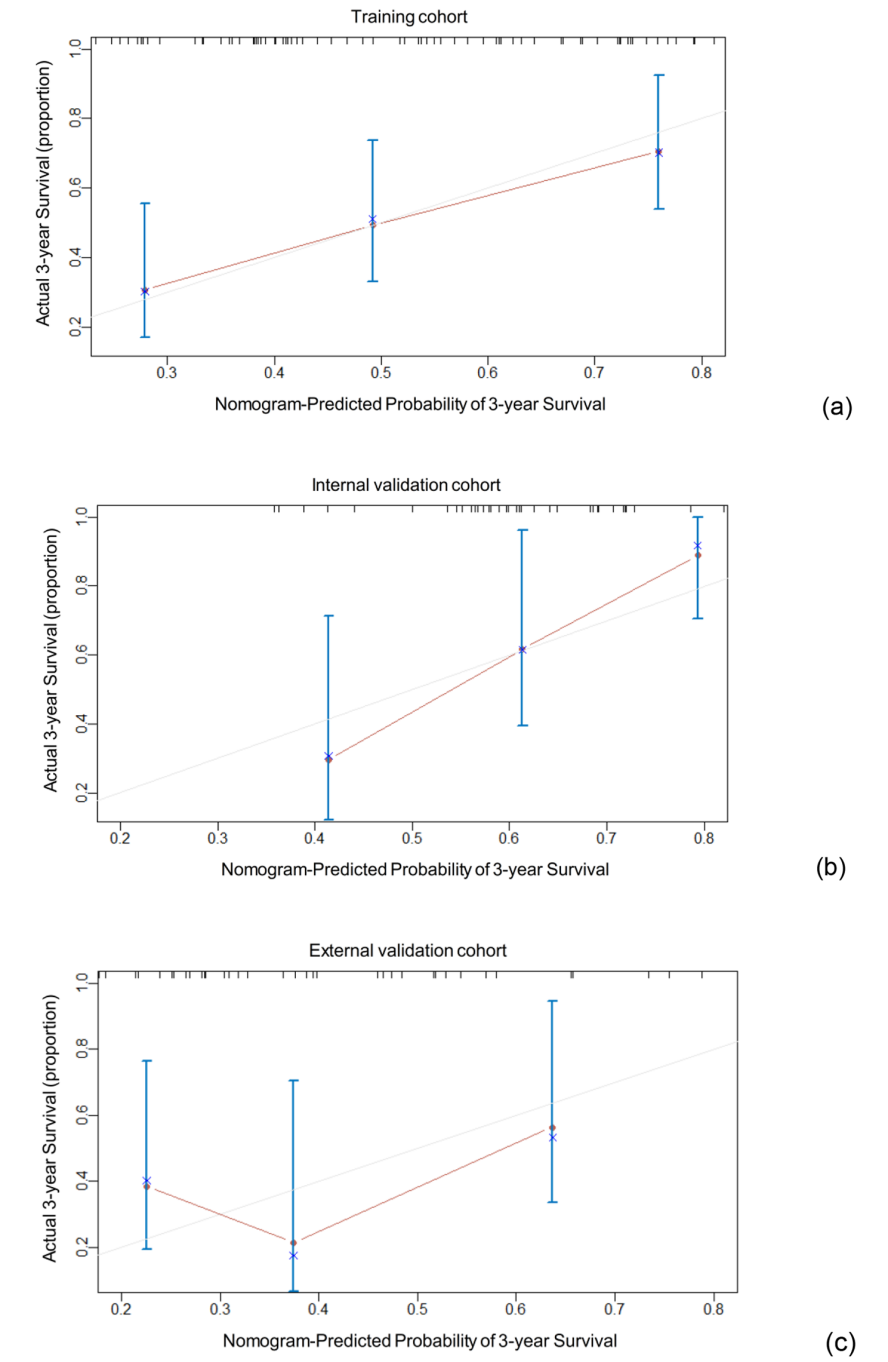
The calibration curve of combined model between the 3-year predicted survival risks and the actual risks in the training (**a**), internal validation (**b**) and external validation cohorts (**c**)

The single CT feature (the tumor thickness), meso-rad-score and t-rad-score were included in the multivariate Cox analysis with Enter method in the training group. The result showed that the meso-rad-score (HR, 134.513; 95% CI, 6.677-2709.755; *p* = 0.001) was the only independent predictors for OS (Table 4). The calibration curve showed good consistency between the 3-year predicted survival risks and the actual risks in the training (Fig. 3a), internal validation (Fig. 3b) and external validation cohorts (Fig. 3c).

**Table 4:** Multivariate Cox regression analysis of models according to the overall survival

	β	HR (95% CI)	*p*
Tumor thickness	0.027	1.027 (0.977-1,080)	0.296
T-rad-score	2.168	8.740 (0.956–79.876)	0.055
Meso-rad-score	4.902	134.513 (6.677-2709.755)	0.001

Note: T−rad−score is for tumoral radiomics score, meso−rad−score is for meso−esophageal fat radiomics score

## Discussion

In this study, we evaluated the capability of the meso-model for prognosis prediction in ESCC patients treated with dCRT. The results revealed that the meso-model had favorable prognostic value in patients from two independent centers. The hereby developed model showed equivalent performance compared to the t-model, and better performance compared to radiological features. Moreover, multivariate analysis showed that the meso-rad-score, but not t-rad-score and radiological feature, was an independent risk factor for OS. The meso-model provided valuable prognostic information for ESCC patients. To our knowledge, this is the first study to explore the prognostic value of radiomic features of meso-esophageal fat in patients with ESCC.

Our results suggested that valuable information about OS was contained in the meso-esophageal fat. Meso-esophagus is an important structure found in the surroundings of the esophagus and contains redundant adipose tissue. The latter is known to manipulate therapeutic resistance by absorbing chemotherapeutic agents and metabolizing and inactivating drugs [28]. Katzmarzyk et al. [29]revealed that visceral adipose tissue was associated with high risk of cancer and cancer-related mortality. Adipocytes remodel stromal cells in the tumor microenvironment via adipokine secretion and act as an energy provider [19, 20]. A previous study on rectal cancer revealed that radiomic features of the mesorectal fat, a tissue around the rectum that is similar to the meso-esophagus, can predict local and distant recurrence [30]. In addition, the meso-esophagus contains blood supply and lymphatic drainage to and from the esophagus [21–23]. It has been found that total meso-esophagus excision reduces regional lymph node recurrence and improves local control of ESCC [24]. However, except for metastatic lymph nodes, the spread of tumor cells in lymphatic vessels throughout the meso-esophagus cannot be visually assessed via routine medical imaging. Our study suggests that the alterations may be detected by radiomics analysis, which is likely to provide more information about the treatment outcome.

In the current study, the meso-model showed equivalent performance compared to the t-model; and multivariate analysis revealed that the meso-rad-score, but not t-rad-score, was an independent risk factor for OS. In terms of prognosis for ESCC patients, the predictive value of the radiomic features extracted from the primary tumors is controversial. A study by Lu et al. showed that the radiomic signature of the assessed tumor was not an independent prognosis factor for OS in ESCC patients who underwent radical esophagostomy [14]. Conversely, in another study, the prognostic aptitude of tumor-based model showed moderate performance for OS in ESCC patients treated by dCRT, with C-indexes of 0.601–0.729 in different cohorts [13].

In our study, the clinical T and N stages were not independent predictive factors for OS. For our ESCC patients, clinical T and N were staged based on CT only. Previous studies revealed that CT has low sensitivity and accuracy in the T stage and nodular metastasis [31]. However, in our case, endoscopic ultrasound and PET-CT, which showed higher accuracy in staging, were available for 48/127 (37.8%) and 85/127 (66.9%) patients in Center 1 and 21/39 (53.8%) and 14/39 (35.9%) patients in Center 2, respectively. Enhanced CT is the most commonly used imaging protocol for staging in clinical settings that is readily available, with low cost, and less operator-dependency. Futhermore, the sample distribution of clinical stage in enrolled patients may be unbalanced and yield biased result in predicting OS. In this study, the patients with clinical stage of IV accounts for 35.7%, 25.6% and 30.7% of all patients in training cohort, internal validation cohort and external validation cohort, respectively. The patients with cT4 account for 32.2%, 20.9% and 30.8% of all patients in training cohort, internal validation cohort and external validation cohort, respectively. However, we focused on investigating meso-esophageal fat in predicting overall survival in this study, and a much larger database of clinical stage IV is needed to predicting OS in esophageal patients in another study. In addition, the result of clinical stage not independently correlated to the OS of the patients is consistent with the previous reports. Previous studies confirmed that patients with early-stage ESCC (cT1N0) had a significantly better outcome than patients with locally advanced ESCC, while there was less difference in the prognosis between patients with locally advanced ESCC. A multicenter study by worldwide esophageal cancer collaboration on 22,123 esophageal cancer patients including 8156 patients with ESCC found that the clinical TNM staging according to the 8th edition of AJCC-UICC TNM staging system had limited prognostic prediction value, especially for locally advanced ESCC treated by esophagectomy [32]. Another study by J. Chen et al. revealed that 3-year survival rates of ESCC based on the 8th edition of cTNM staging system were similar between cT3 and cT4, cN1 and cN2, cN2, and cN3, III and IV stages after definitive chemoradiation or radiotherapy [33]. The results of these studies are similar to our findings, and we suppose that clinical staging has limited predictive value for prognosis for locally advanced ESCC. Radiomics features of meso-esophageal fat complement the insufficient clinical evaluation of OS in patients with ESCC.

This study has clinical implications. While other radiomics studies on ESCC focus on tumor or lymph nodes only, we show that the macro and microenvironment around the tumor have predictive value equivelent to tumor itself for therapeutic outcome evaluation. Furthermore, baseline CT radiomics features of meso-esophageal fat demonstrated better performance compared to clinical and radiological evaluations, but also capability to complement insufficient clinical evaluation before treatment initiation in patients with ESCC to be treated with dCRT.

There are some limitations in the present study. First, this is a retrospective study with small sample size, and selection bias may have affected the results. Second, patients with cervical and abdominal ESCC were excluded. Because the tumoral VOI in the present study covers the entire area of the tumor, and for cervical or abdominal ESCC, chest CT may not completely cover the tumors. Further studies with a wider range of tumoral locations are needed to validate the hereby-developed model. On the other hand, we did not include supplementary imaging methods in the analysis, such as endoscopy ultrasound and PET.

## Electronic supplementary material

Below is the link to the electronic supplementary material.

Supplementary Material 1

## Data Availability

The datasets used and/or analysed during the current study are available from the corresponding author on reasonable request.

## List of Abbreviations

C-index: Concordance Index
dCRT: Definitive chemoradiotherapy
ESCC: Esophageal squamous cell carcinoma
LASSO: Least absolute shrinkage and selection operator
OS: Overall survival
ROC: Receiver operating characteristic
TNM: Tumor, node, metastasis
VOI: Volume of interest
