# Supplementary material for: CT radiomics features of meso-esophageal fat in predicting overall survival of patients with locally advanced esophageal squamous cell carcinoma treated by definitive chemoradiotherapy
Source: BMC Cancer. 2023 May 25;23:477. doi: 10.1186/s12885-023-10973-5 (PMC10210333; doi:10.1186/s12885-023-10973-5)
Supplement: Supplementary file 1 — Supplementary Material 1 [file 12885_2023_10973_MOESM1_ESM.docx]

**Supplement Table 1** CT scanning protocols

|  | Center 1 | Center 2 | |
| --- | --- | --- | --- |
|  | Lightspeed VCT | Somatom Definition AS+ | 256 Revolution |
| Tube current | Automatic based on body weight (50-250mA) | Automatic based on body weight (30-350mA) | Automatic based on body weight (150-550mA) |
| Tube voltage | 120 kVp | 120 kVp | 120 kVp |
| Helical pitch | 0.984 | 0.600 | 0.992 |
| Time for enhanced scan (venous phase) | 55s | 55s-60s | 55s-60s |
| Contrast medium | Iohexol (Omnipaque 300; GE Healthcare) | Ioversol (OptiRay; Guerbet) | Ioversol (OptiRay; Guerbet) |
| Rate of injection | 3.0 mL/s | 2.0-3.0 mL/s | 2.0-3.0 mL/s |
| Dose of contrast medium | 80ml | 85ml | 85ml |

**Supplement Material 1** Criteria of clinical staging used in the present study

Clinically staged T2 ESCC on CT images was seen as definite wall thickening or a mass with a clean outline. cT3 was identified as a tumor with ill-defined abnormal soft tissue around, but with preserved fat planes between the tumor and adjacent structures. cT4 was diagnosed when the tumor invaded the adjacent structures. Pericardial, pleural, or tracheobronchial invasion was suspected when the fat planes between the tumor and the adjacent structures disappeared, or direct extension into the structures was detected. The aortic invasion was suggested if the contact area between the tumor and aorta was greater than 90 degrees(1). Intrathoracic and abdominal lymph nodes with a short-axis diameter larger than 10 mm and supraclavicular lymph nodes with a size larger than 5 mm or heterogeneously enhanced with central necrosis on CT were considered metastatic lymph nodes(1). The number of metastatic lymph nodes confirmed by CT was recorded.

**Supplement Material 2** Quantitative measurements of radiological features

The tumor thickness was measured perpendicularly to the lumen on axial images in locations with a visible lumen. If the lumen was invisible at the slice with the largest tumor, the tumor thickness was measured on the slice on which the lumen was visible and closest to the largest tumor. The tumor length was calculated with slice thickness multiplied by the number of slices in which the tumor was located. Long and short diameters of the largest metastatic lymph nodes were measured on axial images and were recorded as LN-long and LN-short, respectively.

**Supplement Material 3:** A total of 851 features were extracted from the original images including 14 shape features, 18 first-order and 75 texture features derived from gray level co-occurrence matrix (GLCM), gray level dependence matrix (GLDM), gray-level run-length matrix (GLRLM), gray level size zone matrix (GLSZM), and neighborhood gray tone difference matrix (NGTDM), and the same features from the images with the wavelet transform ((18+75)×8＋75＋14=851). Detailed description and definition of the features can be found on <https://pyradiomics.readthedocs.io/en/latest/features.html#>.

**Supplement Table 2** Radiomics features extracted from VOIs

| Classes | Features |
| --- | --- |
| Shape features  (14) | 1. Mesh Volume 2. Voxel Volume 3. Surface Area 4. Surface Area to Volume ratio 5. Sphericity 6. Maximum 3D diameter 7. Maximum 2D diameter (Slice) 8. Maximum 2D diameter (Column) 9. Maximum 2D diameter (Row) 10. Major Axis Length 11. Minor Axis Length 12. Least Axis Length 13. Elongation 14. Flatness |
| First-order features  (18) | 1. Energy 2. Total Energy 3. Entropy 4. Minimum 5. 10th percentile 6. 90th percentile 7. Maximum 8. Mean 9. Median 10. Interquartile Range 11. Range 12. Mean Absolute Deviation (MAD) 13. Robust Mean Absolute Deviation (MAD) 14. Root Mean Squared (RMS) 15. Skewness 16. Kurtosis 17. Variance 18. Uniformity |
| Gray level size zone matrix (GLSZM)  (16) | 1. Small Area Emphasis (SAE) 2. Large Area Emphasis (LAE) 3. Gray Level Non-Uniformity (GLN) 4. Gray Level Non-Uniformity Normalized (GLNN) 5. Size-Zone Non-Uniformity (SZN) 6. Size-Zone Non-Uniformity Normalized (SZNN) 7. Zone Percentage (ZP) 8. Gray Level Variance (GLV) 9. Zone Variance (ZV) 10. Zone Entropy (ZE) 11. Low Gray Level Zone Emphasis (LGLZE) 12. High Gray Level Zone Emphasis (HGLZE) 13. Small Area Low Gray Level Emphasis (SALGLE) 14. Small Area High Gray Level Emphasis (SAHGLE) 15. Large Area Low Gray Level Emphasis (LALGLE) 16. Large Area High Gray Level Emphasis (LAHGLE) |
| Gray Level Co-occurrence Matrix (GLCM)  (24) | 1. Autocorrelation 2. Joint Average 3. Cluster Prominence 4. Cluster Shade 5. Cluster Tendency 6. Contrast 7. Correlation 8. Difference Average 9. Difference Entropy 10. Difference Variance 11. Joint Energy 12. Joint Entropy 13. Informational Measure of Correlation 1 (IMC 1) 14. Informational Measure of Correlation 2 (IMC 2) 15. Inverse Difference Moment (IDM) 16. Maximal Correlation Coefficient (MCC) 17. Inverse Difference Moment Normalized (IDMN) 18. Inverse Difference (ID) 19. Inverse Difference Normalized (IDN) 20. Inverse Variance 21. Maximum Probability 22. Sum Average 23. Sum Entropy 24. Sum of Squares |
| Gray level run-length matrix (GLRLM)  (16) | 1. Short Run Emphasis (SRE) 2. Long Run Emphasis (LRE) 3. Gray Level Non-Uniformity (GLN) 4. Gray Level Non-Uniformity Normalized (GLNN) 5. Run Length Non-Uniformity (RLN) 6. Run Length Non-Uniformity Normalized (RLNN) 7. Run Percentage (RP) 8. Gray Level Variance (GLV) 9. Run Variance (RV) 10. Run Entropy (RE) 11. Low Gray Level Run Emphasis (LGLRE) 12. High Gray Level Run Emphasis (HGLRE) 13. Short Run Low Gray Level Emphasis (SRLGLE) 14. Short Run High Gray Level Emphasis (SRHGLE) 15. Long Run Low Gray Level Emphasis (LRLGLE) 16. Long Run High Gray Level Emphasis (LRHGLE) |
| Gray level dependence matrix (GLDM)  (14) | 1. Small Dependence Emphasis (SDE) 2. Large Dependence Emphasis (LDE) 3. Gray Level Non-Uniformity (GLN) 4. Dependence Non-Uniformity (DN) 5. Dependence Non-Uniformity Normalized (DNN) 6. Gray Level Variance (GLV) 7. Dependence Variance (DV) 8. Dependence Entropy (DE) 9. Low Gray Level Emphasis (LGLE) 10. High Gray Level Emphasis (HGLE) 11. Small Dependence Low Gray Level Emphasis (SDLGLE) 12. Small Dependence High Gray Level Emphasis (SDHGLE) 13. Large Dependence Low Gray Level Emphasis (LDLGLE) 14. Large Dependence High Gray Level Emphasis (LDHGLE) |
| Neighborhood gray tone difference matrix (NGTDM)  (5) | 1. Coarseness 2. Contrast 3. Busyness 4. Complexity 5. Strength |

**Supplement** **Table3** Univariate and multivariate Cox regression analysis of clinical and radiological findings according to the overall survival

|  | Univariate analysis | | | Multivariate analysis | | | | |
| --- | --- | --- | --- | --- | --- | --- | --- | --- |
|  | HR (95% CI) | *p* | | β | HR (95% CI) | | *p* | |
| Age | 0.998 (0.972-1.024) | 0.853 | |  |  | |  | |
| Sex |  | 0.213 | |  |  | |  | |
| Male | Reference |  | |  |  | |  | |
| Female | 0.658 (0.341-1.271) |  | |  |  | |  | |
| BMI | 1.013 (0.943-1.088) | 0.722 | |  |  | |  | |
| Clinical T stage^#^ |  | 0.954 | |  |  | |  | |
| T2~3 | Reference |  | |  |  | |  | |
| T4 | 0.986 (0.616-1.578) |  | |  |  | |  | |
| Clinical N stage^#^ |  | 0.217 | |  |  | |  | |
| N0 | Reference |  | |  |  | |  | |
| N1 | 1.188 (0.576-2.449) |  | |  |  | |  | |
| N2 | 1.771 (0.888-3.530) |  | |  |  | |  | |
| N3 | 1.767 (0.696-4.491) |  | |  |  | |  | |
| Clinical stage^#^ |  | 0.852 | |  |  | |  | |
| II~III | Reference |  | |  |  | |  | |
| IV | 1.043 (0.666-1.634) |  | |  |  | |  | |
| Tumor thickness | 1.033 (1.004-1.063) | 0.025^*^ | | 0.033 | 1.033 (1.004-1.063) | | 0.025^**^ | |
| Tumor length | 1.011 (0.999-1.022) | 0.064^*^ | |  |  | |  | |
|  |  |  | |  |  | |  | |
|  |  |  | |  |  | |  | |
| No. of metastatic LN^#^ | 1.052 (0.987-1.121) | 0.121 | |  |  | |  | |
| LN-long | 1.001 (0.983-1.019) | 0.938 | |  |  | |  | |
| LN-short | 1.001 (0.978-1.024) | 0.958 | |  |  | |  | |
|  |  |  | |  |  | |  | |
|  |  |  | |  |  | |  | |

^Note: # The clinical stage of ESCC and no. of metastatic lymph node were based on the evaluation of contrast-enhanced CT images. LN is for lymph node, LN-long is for long diameter of the lymph node, LN-short is for the short diameter of the lymph node, * was marked as^ *^p^* ^<0.10, ** marked as^ *^p^* ^<0.05.^

**Supplement Material 3 Tumor-based radiomics score**

T-rad-score= 0.0004550102 × original_glrlm_RunLengthNonUniformity

+ 0.0030743464 × original_shape_MajorAxisLength

- 7.5627236958 × wavelet.HHH_glcm_SumEntropy

+ 0.1157376512 × wavelet.LHH_glszm_ZoneEntropy

1. Hong SJ, Kim TJ, Nam KB, Lee IS, Yang HC, Cho S et al. New tnm staging system for esophageal cancer: What chest radiologists need to know. Radiographics 2014;34:1722-1740
